# Enzymatically-Processed Wheat Bran Enhances Macrophage Activity and Has *in Vivo* Anti-Inflammatory Effects in Mice

**DOI:** 10.3390/nu8040188

**Published:** 2016-04-01

**Authors:** Hee Kang, Mi-Gi Lee, Jae-Kang Lee, Yong-Hyun Choi, Yong-Seok Choi

**Affiliations:** 1 Department of East-West Medical Science, Graduate School of East-West Medical Science, Kyung Hee University, Yongin 17104, Korea; 2 Bio-Center, Gyeonggi Institute of Science and Technology Promotion, Suwon 16229, Korea; migi@gstep.re.kr; 3 DongA One Corporation, Dangjin 31703, Korea; macmen@kodoco.com (J.-K.L.); camdol@kodoco.com (Y.-H.C.); knownet@kodoco.com (Y.-S.C.)

**Keywords:** wheat bran, arabinoxylan, functional food, macrophage, inflammation

## Abstract

Wheat bran is a rich source of dietary fiber, of which arabinoxylan is the most abundant non-starch polysaccharide. Arabinoxylan has been known to exert *in vivo* immunological activities. Based on prior findings, we pretreated wheat bran with enzymatic hydrolysis to increase the release of soluble arabinoxylan and investigated whether oral administration of wheat bran altered macrophage activity in a mouse model. After four weeks of treatment, we isolated peritoneal macrophages for phagocytic receptor analysis and lipopolysaccharide (LPS)-induced inflammatory changes. In the second experiment, mice given wheat bran were intraperitoneally stimulated with LPS and serum levels of pro- and anti-inflammatory cytokines were determined. The expression of SRA and CD36, and phagocytic activity increased (*p* < 0.05, respectively). *Ex vivo* stimulation of macrophages by LPS resulted in reduced surface expression of CD40 (*p* < 0.05) and decreased production of nitric oxide (*p* < 0.005), tumor necrosis factor (TNF)-α (*p* < 0.005), interleukin (IL)-6 (*p* < 0.01), and IL-12 (*p* < 0.05). Mice treated with wheat bran showed decreased levels of serum TNF-α and IL-6 (*p* < 0.05, respectively) and an increased level of serum anti-inflammatory IL-10 (*p* < 0.05) in response to intraperitoneal LPS. Enzymatically-processed wheat bran boosts macrophage phagocytic capacity possibly through up-regulation of scavenger receptors and confers anti-inflammatory effects, indicating its potential as an immuno-enhancing functional food.

## 1. Introduction

Wheat bran is the outermost layer of the wheat grain, representing 14% to 19% of the total weight [1]. Wheat bran is a rich source of dietary fiber, such as non-starch polysaccharides and lignin. Arabinoxylan accounts for 30% of dry wheat bran mass, or 70% of non-starch polysaccharides [1]. It is a polymer with a d-xylose backbone linked with l-arabinose. Phenolic compounds, such as ferulic acid, are attached to the arabinose residue. Most arabinoxylan in wheat bran is water insoluble because of crosslinking with neighboring units of cellulose, lignin, and proteins [2]. Since it is rich in insoluble fibers, which are slowly fermented in the intestine [3], wheat bran is beneficial in producing bulky stool and preventing colon cancer [4,5]. In addition, wheat bran has been reported to decrease some of the inflammatory markers associated with obesity in mice fed a high-fat diet [6].

Innate immunity is characterized by a rapid response to invading pathogens, but lacks memory function, whereas adaptive immunity requires several days to mount a response during its first encounter with an antigen, but acquires memory. Evidence is accumulating that polysaccharides from certain plants have immunological activity. A study reviewing the therapeutic potential of plant polysaccharides indicated that most research has focused on modulating innate immunity, specifically macrophages [7]. Macrophages are the first line of a host’s innate immune system defense. They detect the presence of foreign organisms that have penetrated physical and chemical barriers. Macrophages are phagocytes with pattern recognition receptors (PRRs) that recognize foreign ligands with “pathogen-associated molecular patterns”. PRRs are involved in either phagocytosis or inflammatory response signaling [8]. Phagocytic receptors, such as scavenger receptors, lectin-type receptors, Fc receptors, and complement receptors, internalize target substances by forming phagosomes that digest the captured targets. On the other hand, signaling receptors such as toll-like receptors (TLR) initiate the inflammatory response through NF-κB activation, which results in the production of bactericidal substances such as reactive oxygen species and nitric oxide (NO) or the secretion of pro-inflammatory cytokines such as tumor necrosis factor (TNF)-α, interleukin (IL)-1, IL-6, and IL-12. At the same time, TLR-activated macrophages produce an anti-inflammatory cytokine IL-10 [9]. IL-10 is able to regulate the production of TNF-α. Macrophages can also activate T helper (Th) cells in a cell-to-cell contact mode. Inflammatory stimuli induce CD40 and CD80/CD86, called costimulatory molecules, on the macrophages. They make contact with ligands on the Th cells to provide sufficient signals between cells. The mechanism underlying the activation of macrophages by plant-derived polysaccharides might be mediated by the same PRR, which recognizes them and begins inflammatory signaling cascades [7]. However, most such effects have emerged from *in vitro* or *in vivo* studies in which polysaccharides were non-orally administered [10].

Recently, Zhou *et al.* compared and evaluated the immunological activities of wheat bran arabinoxylan extracted using an alkaline solution or enzyme, both of which are common methods for extracting insoluble arabinoxylan from complex plant materials [11]. They showed that enzyme-derived arabinoxylan was more potent in macrophage phagocytosis and T cell-dependent macrophage response than alkaline-aided arabinoxylan. Enzymatically-processed arabinoxylan contains a higher ratio of xylose to arabinose (a pattern similar to that found in water-extractable arabinoxylan) than alkaline-extracted arabinoxylan (which shows a similar ratio of xylose to arabinose as water-unextractable arabinoxylan [1,11]. Based on prior study, we seek to investigate whether oral consumption of wheat bran, itself, shows a similar immunoenhancing activity. Given safety issues and for the application of wheat bran as a functional food, enzymatic treatment is the preferred choice for arabinoxylan extraction. We treated wheat bran with xylanases, which break random β-1,4-glycosyl linkages within the xylose backbone and make the water-insoluble arabinoxylan embedded in wheat bran easily solubilized. Then we administered mice with this enzymatically-processed wheat bran and examined its potential immunomodulatory effects on macrophage activity and inflammatory responses.

## 2. Experimental Methods

### 2.1. Preparation of Enzyme-Treated Wheat Bran

Wheat bran was supplied by DongA One (Dangjin, Korea). Due to the complexity of its organic structure, we first pretreated it using extrusion (heating, compression, shear, and swelling). The wheat bran was processed in a twin extruder (FX40, Milling Industry, Seoul, Korea) with a length-to-diameter ratio (L/D) of 17:1 and a die-hole diameter of 3 mm. The extrusion conditions were a barrel temperature of 100 °C, 15% moisture content, and a screw speed of 550 rpm. After extrusion, three grams of extruded wheat bran was mixed with 30 mL of deionized water (DW) in a 50 mL conical tube. The suspension was heated in an autoclave at 121 °C for 1 h, then cooled to 40–50 °C. An amount of enzyme (Celluclast 1.5 L, Novozymes, Denmark) corresponding to 2% (*w*/*w*) of the extruded wheat bran was added to the suspension while vortexing for 2 h. The mixture was ground in a mill (Laboratory Mill 3100, Perten, Finland) after drying in a drum dryer (D-0303, Katsuraki, Japan).

### 2.2. Determination of Dietary Fiber Content

The amounts of total dietary fiber (TDF), insoluble dietary fiber (IDF), and soluble dietary fiber (SDF) were determined following the enzymatic-gravimetric method given in the Association of Analytical Chemists 991.43 [12]. Samples were ground fine enough to pass through a 0.5-mm screen, gelatinized with heat-stable α-amylase (95 °C for 30 min), then digested with protease (60 °C for 30 min) and amyloglucosidase (60 °C for 30 min) to remove starch and protein. To weigh the TDF, the digested sample was filtered and the residue was washed with 78% ethanol, 95% ethanol, and acetone in turn. For the analysis of IDF, the digested sample was filtered and the residue with 60 °C DW to precipitate the IDF. The filtrate and the washed water obtained above were combined and 95% ethanol was added to precipitate the SDF. All of the residues obtained were weighed after drying at 105 °C in a hot air oven. TDF, IDF, and SDF values were corrected for protein and ash content.

### 2.3. Determination of Water Soluble Arabinoxylan

Water soluble arabinoxylan content was determined based on a colorimetric method described by Douglas *et al.* [13]. The method hydrolyzes arabinoxylan to its constituent pentose sugars, which are then reacted with phloroglucinol. Twenty-five milligrams of each sample and 25 mL of DW were added to each test tube. After shaking the tubes for 30 min and centrifuging them, 1 mL of the supernatant was transferred to a new tube and 1 mL DW was added to bring the total volume to 2 mL. Then 10 mL of the reaction reagent (110 mL glacial acetic acid, 30 mL hydrochloric acid, 60 mL of 20% *w*/*v* phloroglucinol in ethanol, and 1 mL of 1.75% *w*/*v* glucose in water) was added to each tube. The tubes were then placed in a boiling water bath for 25 min, cooled in an ice bath, and moved to a room-temperature bath. Absorbance readings at 552 and 510 nm were calibrated against a xylose standard using a spectrophotometer (Libra S22, Biochrom, Cambridge, UK) and the results were expressed as a percentage by weight.

### 2.4. Analysis of Sugar Composition

High-performance liquid chromatography (HPLC) analysis of sugar monomers was determined using laboratory analytical procedures proposed by the National Renewable Energy Laboratory [14]. Samples were hydrolyzed in sealed tubes containing 100 μL of trifluoroacetic acid at 100 °C for 4 h. After digestion, the pH was adjusted to 6.0 by adding 300 μL of high purity water and each sample was filtered using a 0.2-μm syringe filter. All samples were analyzed using a Dionex HPLC system equipped with a Dionex Carbopac PA1 column and electrochemical detector (HPAEC-PAD system, Dionex, Sunnyvale, CA, USA). Dilute NaOH solution (18 mM) was used as the eluent, with a constant flow rate of 1.0 mL/min. Column temperature was maintained at 36 °C. Fifty microliters of sample was mixed with 450 μL of high purity water (*i.e.*, 1:10 dilution) in an injection vial. The injection volume was 25 μL. Reagent grade d(−)arabinose, d(+)-galactose, d(+)-glucose, and d(+)-xylose (all Sigma, St. Louis, MO, USA) were used to prepare 2, 20, 100, and 200 μM standards. Data for these standards were used to construct separate calibration curves for each monomer. Selected samples were also spiked with known concentrations of arabinose, galactose, glucose, and xylose to confirm peaks in the HPLC analyses.

### 2.5. Animals

Seven-week-old male Balb/c mice were obtained from SamTaco (Osan, Korea) and housed in a temperature- and humidity-controlled, pathogen-free animal facility with a 12-h light-dark cycle. Our animal protocol (KHUASP(SE)-15-012) was approved by the Kyung Hee University Institutional Animal Care and Use Committee, and mice were cared for according to the US National Research Council for the Care and Use of Laboratory Animals (1996) specifications.

### 2.6. In Vivo Experiment 1

Mice were divided into three groups (control, 500 mg/kg, and 2500 mg/kg, *n* = 10). Enzymatically-processed wheat bran was suspended in water and given via oral gavage once daily for four weeks. The control group was given an equal amount of water. The body weight of each group was monitored every week and there was no difference among groups during the experimental period. Four days prior to sacrifice, mice were injected intraperitoneally with 2 mL of 3.5% sterile thioglycollate solution (BD, Sparks, MD, USA). At the end of the treatment, mice were sacrificed by cervical dislocation and peritoneal macrophages were isolated by peritoneal lavage with cold DMEM. After centrifugation, cells were re-suspended in DMEM with 10% fetal bovine serum (FBS; Hyclone, Logan, UT, USA) and 1% penicillin-streptomycin. We then used the cells for flow cytometry assays or *ex vivo* culture.

### 2.7. iExperiment 2

Mice were given a dose of 2500 mg/kg once daily for four weeks (*n* = 12). At the end of the experimental period, 1.3 mg/ kg LPS (serotype 055:B5, Sigma) was intraperitoneally injected. One hour later blood was collected intraorbitally and centrifuged. The obtained serum was stored at −20 °C until assay.

### 2.8. Flow Cytometry

Cells were washed twice in phosphate buffered saline (PBS) and re-suspended at 1 × 10^6^ cells/mL in FACS staining buffer (PBS/0.1% NaN_3_/1% FBS). Cells were blocked with rat anti-mouse CD16/CD32 (BD Pharmingen, San Diego, CA, USA) at 4 °C for 5 min and then stained for 30 min with FITC-conjugated anti-mouse SRA, anti-mouse CD11b, anti-CD40, PE-conjugated anti-mouse CD11b, anti-mouse CD36, and anti-mouse CD86 (all BD Pharmingen) on ice in the dark. The cells were washed, re-suspended and analyzed on a Navios Flow Cytometer (Beckman Coulter, Brea, CA, USA). The data were processed using Kaluza software.

### 2.9. Cell Culture Ex Vivo

Cells were plated overnight at 37 °C, and non-adherent cells were removed. Cells were stimulated with 100 ng/mL LPS for 24 h. Supernatant and cells were collected for subsequent assays.

### 2.10. Phagocytosis Assay

Cells were cultured with FITC-immunoglobulin (Ig)-latex beads (Cayman Chemical, Ann Arbor, MI, USA) for 24 h and then collected for flow cytometry analysis.

### 2.11. Nitric Oxide Determination

Fifty microliters supernatant obtained from the above culture was incubated with an equal volume of Griess reagent (Sigma) for 15 min at room temperature. We measured the absorbance at 550 nm with a microplate reader (Molecular Devices, Sunnyvale, CA, USA).

### 2.12. Cytokine Analysis

We determined levels of TNF-α, IL-6, IL-10 and IL-12 in supernatant or serum by enzyme-linked immunosorbent assay (ELISA) according to the manufacturer’s protocol (BD Pharmingen).

### 2.13. Statistical Analysis

Data were analyzed by student *t* test or ANOVA followed by Tukey *post hoc* test using IBM SPSS software (version 22, IBM, Chicago, IL, USA). *p* values less than 0.05 were considered significant.

## 3. Results

### 3.1. Analysis of Dietary Fiber, Arabinoxylan, and Monosaccharides

We analyzed the wheat bran for dietary fiber, water soluble arabinoxylan, and monosaccharides. Table 1 shows the chemical composition of untreated wheat bran, extruded wheat bran, and enzymatically-processed wheat bran (wheat bran with combination of extrusion and enzyme treatment). The methods used here for the evaluation of dietary fiber did not include oligosaccharides released by xylanases and, thus, the content of insoluble dietary fiber and total dietary fiber in the enzymatically-processed wheat bran decreased from 39.44% and 43.75% to 30.84% and 38.65%, respectively (Table 1). Extrusion, alone, increased the amount of soluble arabinoxylan by 2.2-fold, and a combination of extrusion and enzyme treatment increased it 22-fold. Component sugar analysis showed that enzymatically-processing wheat bran increased the release of xylose and arabinose two-fold (Table 2).

### 3.2. Effect of Enzymatically-Processed Wheat Bran on Scavenger Receptor Expression and Phagocytic Activity in Macrophages

**Figure 1:**
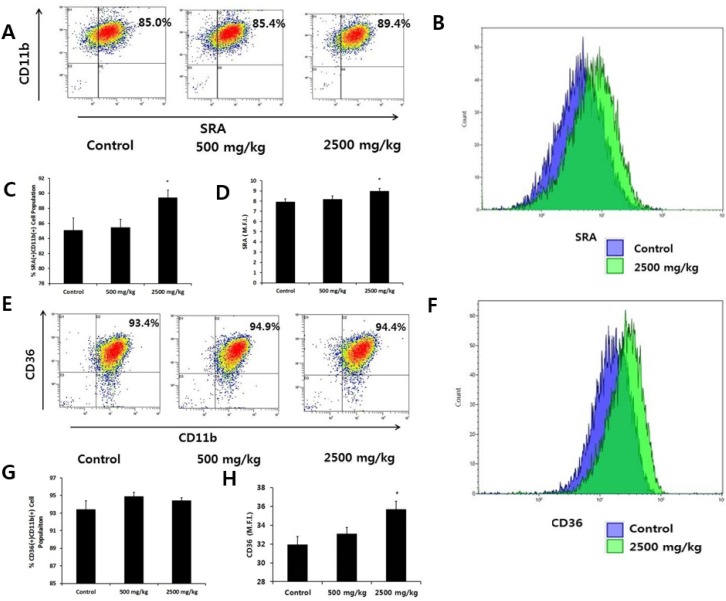
Scavenger receptors expressed by macrophages after oral administration of enzymatically-processed wheat bran. Peritoneal macrophages were isolated after four weeks of oral administration of enzymatically-processed wheat bran (500 or 2500 mg/kg). The cells were stained for FITC-conjugated SRA antibody (ab) and PE-conjugated CD36 ab, or FITC-conjugated CD11b ab and PE-conjugated CD36 ab. (**A**,**E**): we analyzed the percentages of the SRA(+)CD11b(+) or CD36(+)CD11b(+) cell populations using flow cytometry and show representative dot plots; (**B**,**F**): we analyzed the mean fluorescence intensity (M.F.I.) of SRA or CD36 using flow cytometry and show representative histograms. Blue and green lines depict the control and 2500 mg/kg groups, respectively; (**C**,**D**,**G**, and **H**): Bars represent mean ± SEM of data (*n* = 5). * *p* < 0.05 *versus* control.

**Figure 2:**
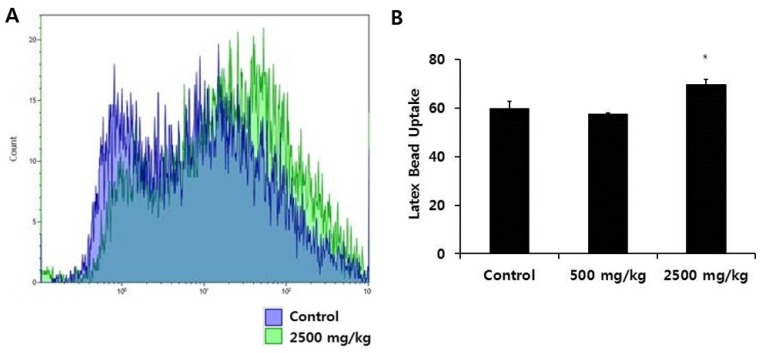
Effect of enzymatically-processed wheat bran on phagocytic ability of macrophages. We incubated peritoneal macrophages from each group with FITC-immunoglobulin (Ig)-latex beads for 24 h and analyzed the uptake of latex beads by flow cytometry. (**A**): representative histograms are shown. Blue and green lines depict control and 2500 mg/kg groups, respectively; (**B**): bars represent mean ± SEM (*n* = 5). * *p* < 0.05 *versus* control.

We first measured whether enzymatically-processed wheat bran influenced the levels of SRA and CD36, representative macrophage scavenger receptors. We used CD11b as a marker for macrophages and found that more than 99% of peritoneal macrophages were CD11b(+). SRA and CD36 molecules were expressed only in CD11b(+) cells (Figure 1A,E). Comparing the percentage of SRA(+)CD11b(+) cells and the mean fluorescence intensity (M.F.I.) of SRA, indicative of the amount of SRA bound to FITC-conjugated antibodies, among groups, we observed a significant increase in the high-dose group (Figure 1A–D). With regard to CD36 molecules, we found no difference in CD36(+)CD11b(+) cells among the groups, but the M.F.I. value of CD36 increased significantly in the high-dose wheat bran group (Figure 1E–H). We then determined the intracellular uptake of FITC-Ig-latex beads for the evaluation of phagocytosis. Since the latex beads used here were coated with Ig, the phagocytic receptor responsible for the internalization of those particles was the Fc receptor. We cultured peritoneal macrophages from each group with FITC-Ig-latex beads for 24 h. The M.F.I. value of the high dose group increased significantly, indicating an enhanced phagocytic capacity compared with the control group (Figure 2).

### 3.3. Effect of Enzymatically-Processed Wheat Bran on NO and Cytokines in LPS-Stimulated Macrophages

**Figure 3:**
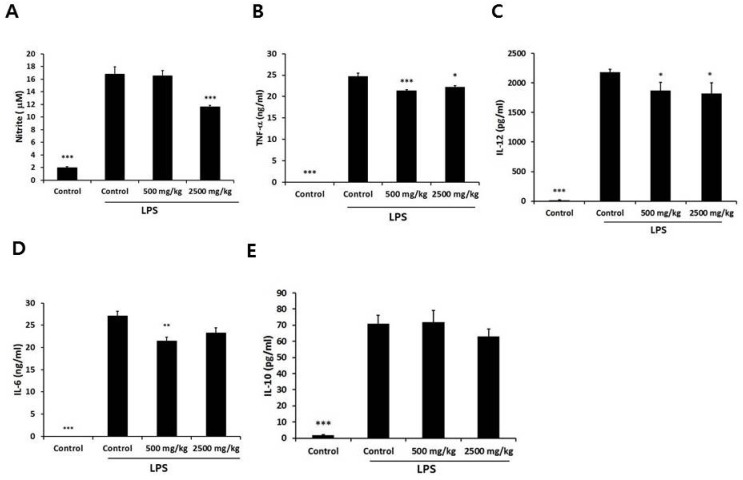
Effect of oral administration of enzymatically processed wheat bran on nitric oxide and soluble pro-inflammatory and anti-inflammatory cytokines in LPS-stimulated macrophages. Macrophages isolated from each group were stimulated with LPS (100 ng/mL) for 24 h. (**A**): the level of nitric oxide was measured by nitrite colorimetric assay; (**B**–**E**): the levels of tumor necrosis factor (TNF)-α, interleukin (IL)-12, IL-6, and IL-10 were determined by ELISA. Data represent mean ± SEM (*n* = 5). * *p* < 0.05, ** *p* < 0.01, *** *p* < 0.005 *versus* LPS-treated control group.

We sought to determine whether oral administration of enzymatically-processed wheat bran influences the inflammatory response of macrophages. Therefore, peritoneal macrophages isolated from the control and enzymatically-processed wheat bran groups were stimulated with LPS, a TLR4 agonist, for 24 h. We determined the level of NO by measuring the level of nitrite, a stable metabolite during nitric oxide reactions, because the half-life of NO is less than 15 s [15]. The high-dose group showed a significant reduction in NO of 31% compared with the control group (Figure 3A). We also measured soluble pro-inflammatory and anti-inflammatory cytokines. With respect to the pro-inflammatory cytokines, the levels of LPS-stimulated TNF-α and IL-12 in the supernatant were significantly suppressed in both the low-and high-dose groups, with the reduction of TNF-α in the low-dose group being more marked (Figure 3B,C). The levels of LPS-stimulated IL-6 in the supernatant were also suppressed, but those of the low-dose group were significant (Figure 3D). However, there was no significant difference in IL-10 (Figure 3E).

### 3.4. Effect of Oral Administration of Enzymatically-Processed Wheat Bran on Contact Dependent Inflammatory Molecules in LPS-Activated Macrophages

**Figure 4:**
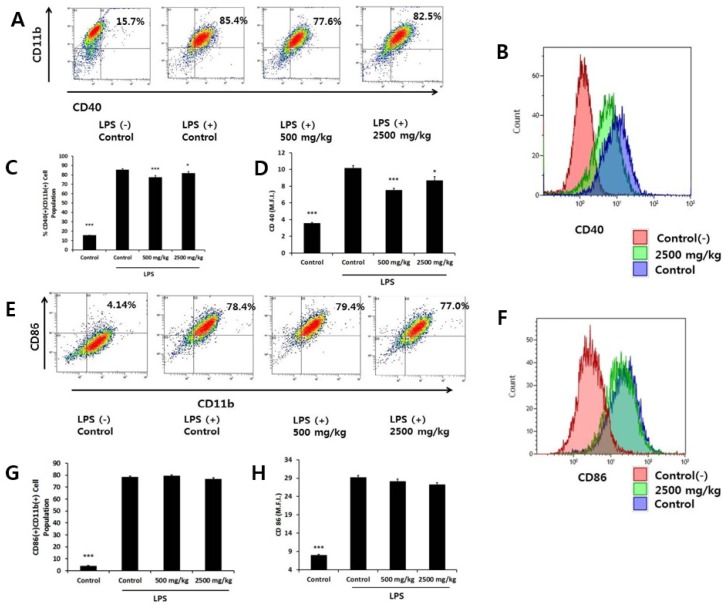
Regulation of contact dependent inflammatory molecules expressed by LPS-stimulated macrophages after oral administration of wheat bran. Cells isolated from each group were stimulated with LPS for 24 h. The cells were stained for FITC-conjugated CD40 antibody (ab) and PE-conjugated CD11b ab or FITC-conjugated CD11b ab and PE-conjugated CD86 ab. (**A**,**E**): we analyzed the percentages of the CD40(+)CD11b(+) or CD86(+)CD11b(+) cell population using flow cytometry and show representative dot plots; (**B**, **F**): we analyzed the M.F.I. of CD40 or CD86 using flow cytometry and show representative histograms of the control and 2500 mg/kg groups. Red, blue, and green lines depict the control (−), control and 2500 mg/kg groups, respectively; (**C**,**D**,**G**,**H**): bars represent mean ± SEM of data (*n* = 5). * *p* < 0.05, *** *p* < 0.005 *versus* LPS treated control group.

An important feature of macrophage function is to present antigens to Th cells through direct cell-to-cell contact. We stimulated macrophages with LPS and determined the surface expression of CD40 and CD86 molecules by flow cytometry. Stimulation with LPS increased the percentages of CD40(+)CD11b(+) cells and CD86(+)CD11b(+) cells from 15.7% to 85.5% and from 4.1% to 78.4%, respectively (Figure 4A,E). The low- and high-dose groups both significantly decreased the percentage of CD40(+)CD11b(+) cells, with an accompanying decrease in the CD40 M.F.I. value (Figure 4A–D). On the other hand, we found no difference in the percentage of CD86(+)CD11b(+) cells or the CD86 M.F.I. value among groups (Figure 4E–H).

### 3.5. Effect of Enzymatically-Processed Wheat Bran on Serum Cytokines in Response to Intraperitoneal Injection of LPS

**Figure 5:**
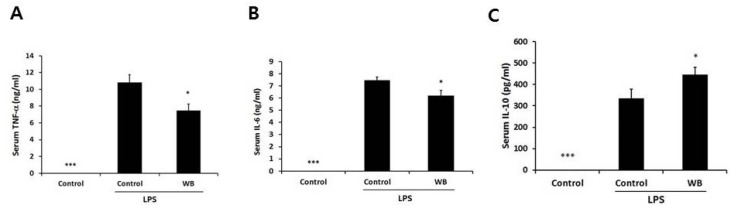
Effect of wheat bran on serum inflammatory responses following LPS injection. Mice were orally given enzymatically-processed wheat bran (WB) for four weeks. Serum was obtained 1 h after intraperitoneal injection of LPS (1.3 mg/kg). We measured the serum levels of TNF-α, IL-6 and IL-10 by ELISA. Data represent mean ± SEM (*n* = 12). * *p* < 0.05, *** *p* < 0.005 *versus* control.

Since the above results demonstrated that macrophages from mice treated with enzymatically-processed wheat bran showed anti-inflammatory activity *ex vivo*, it is important to confirm whether such anti-inflammatory responses occur *in vivo*. After mice were given a dose of 2500 mg/kg for four weeks, we intraperitoneally injected the mice with a sublethal dose of LPS and then measured the levels of serum TNF-α, IL-6, IL-12, and IL-10 at 1 h after LPS challenge. As the peak responses of these cytokines *in vivo* were different [16], TNF-α, IL-6, and IL-10 were the only detectable cytokines. Enzymatically-processed wheat bran decreased the levels of TNF-α and IL-6, as seen with *ex vivo* culture (Figure 5). On the contrary, serum IL-10 was significantly increased, suggesting that different cell sources other than macrophages may respond to wheat bran. These data demonstrate that enzymatically-processed wheat bran is able to systematically down-regulate the acute inflammatory responses.

## 4. Discussion

Since wheat is the most widely grown cereal in the world, wheat bran is produced abundantly during the milling process. Wheat bran is a rich source of antioxidant phytochemicals and dietary fiber. Given that its primary use is limited to the animal feed industry, attempts to develop wheat bran as a functional food are cost-effective and environmentally friendly. As it is high in dietary fiber, wheat bran is an ideal addition to a variety of food products for individuals whose consumption of dietary fiber is low. In addition, the antioxidant activity of wheat, which is mostly ascribed to its phenolics and fiber, is largely found in the bran layer [17,18]. To explore the value of wheat bran, we present here the immune-enhancing potential of a crude form of wheat bran, which is minimally processed and, thus, cost efficient.

The significance of macrophage phagocytosis is that macrophages eliminate not only invading foreign organisms, but also dead cells, debris released by dying cells, and unwanted tissue components originating from the host. Such clearance should be rapid to prevent inflammatory responses [19]. Scavenger receptors are particularly abundant in macrophages and recognize a variety of foreign and host-negative charge macromolecules, such as bacterial LPS and oxidized lipoproteins [20]. Oral treatment with enzymatically-processed wheat bran enhanced the expression of SRA and CD36 molecules, suggesting an improved capacity for phagocytosis. In addition, Fc receptors, another type of phagocytic receptor, internalize an antigen/antibody complex by binding to the Fc portion of an antibody, allowing macrophages to take part in the biological function of adaptive immunity [21]. The increased uptake of FITC-Ig-latex beads by cells from mice fed enzymatically processed wheat bran indicates that the activity or expression of the Fc receptor was enhanced. Overall, enzymatically-processed wheat bran boosted the phagocytic activity of macrophages, possibly via up-regulation of phagocytic receptors.

NO is synthesized from l-arginine by NO synthases in the presence of oxygen. Among the three isoforms of NO synthase, type 2 NO synthase, or inducible NO synthase in macrophages is expressed by pathogens or microbial products and interferons [22]. Although NO is antimicrobial, its exaggerated production is detrimental to host cells. TNF-α, IL-6 and IL-12 are important because at high enough concentrations, they restrict the spread of pathogens and attract blood immune cells to a site where pathogens have not been entirely removed by macrophages. IL-12 specifically affects the differentiation of T helper type I cells, which subsequently activates macrophages [23]. Despite the importance of these local soluble inflammatory mediators, inappropriate production of them by macrophages damages neighboring tissue. We found that macrophages from the enzymatically processed wheat bran group showed anti-inflammatory activity *ex vivo*, as seen by the reduced levels of NO, TNF-α, IL-6 and IL-12. Inducible NO synthase, TNF-α, and IL-6 are all produced by the LPS/TLR4-mediated NF-κB and mitogen-activated protein (MAP) kinase protein signaling pathways [24]. We did not explore how these intracellular signaling molecules were affected.

CD40 is a cell surface molecule that mediates contact-dependent intracellular signaling, enhancing not only T cell response, but also a macrophage’s own function, such as the synthesis of NO, TNF-α, and IL-6 and the up-regulation of co-stimulatory molecules, such as CD86 and CD40 itself [25]. CD40 expression in macrophages is increased by TLR agonists through NF-κB and MAP kinases [26]. Blocking CD40 signaling at the cell surface is reported to inhibit various chronic inflammatory and autoimmune disorders [25]. Notably, the pattern we observed in the down-regulation of LPS-induced CD40 expression was similar to that observed in TNF-α and IL-6, with the low-dose group seeing more effect than the high-dose group. We speculate that with rising dose, other unidentified components override the specific inhibitory effects seen on CD40, TNF-α, and IL-6 at the low dose. CD86 is involved in the presentation of antigens to Th cells and is used as a marker for activated macrophages [27,28]. This molecule was not suppressed by the treatment of enzymatically-processed wheat bran, indicating that CD86 and CD40 are differentially regulated.

We demonstrated that the anti-inflammatory effect of enzymatically-processed wheat bran occurred in response to systemic injection of LPS. As seen with the *ex vivo* data, the levels of pro-inflammatory cytokines TNF-α and IL-6 in serum were decreased. Interestingly, the level of anti-inflammatory IL-10 was significantly increased in serum from the wheat bran group, which was not evident in LPS-treated macrophages. Since IL-10 is produced not only by macrophages but also by other innate and adaptive immune cells [9], the induction of IL-10 by wheat bran may extend beyond macrophages.

Prebiotics are defined as food ingredients that resist gastric acidity, hydrolysis by host enzymes, and gastrointestinal absorption; are fermented by intestinal bacteria; and boost human health by selectively stimulating the growth or activity of beneficial intestine bacteria, specifically *Bifidobacterium* and *Lactobacillus* species [2]. Arabinoxylan, and its hydrolysis product arabinoxylan-oligosaccharides (AXOS), meet the criteria for prebiotics [2]. Arabinoxylan itself is not a direct substrate fermented by *Bifidobacterium*. Intestinal arabinoxylan is fermented first by rumen *Prevotella* spp. or human colonic *Bacteroides* spp., and then its hydrolysis products, such as xylooligosaccharides and AXOS, are fermented by *Bifidobacterium*, *Lactobacillus*, and *Bacteroides*. According to randomized, placebo-controlled human studies [29,30,31,32,33], oral intake of wheat bran extract or arabinoxylan-oligosaccharides increased stool *Bifidobacterium* species and short chain fatty acid production and decreased colonic protein fermentation, which led to the reduced formation of potentially harmful metabolites such as nitrogen-containing compounds and thiols. The health promoting effects of bifidobacteria in humans have been demonstrated by reducing the risk of respiratory infections and ameliorating symptoms related to irritable bowel syndrome [34,35]. The mechanisms of how bifidobacteria improve such effects or influence the resident host microbiota have not yet been determined but evidence is accumulating that alterations in gut microbiota modulate the immune system [36]. In support of this, intraperitoneal treatment of arabinoxylan purified from wheat bran did not show any immunostimulating effect, unlike oral treatment, suggesting that its effect could be related to its metabolism in the gut [11]. Therefore, the potential prebiotic effects of enzymatically-processed wheat bran might mediate its immuno-enhancing and anti-inflammatory effects. In addition, the effect of antioxidant components within enzymatically processed wheat bran should not be excluded. The bran layer is a rich source of phenolic acids, particularly ferulic acid [18]. Enzymatic treatments enhance the release of ferulic acid and other phenolic acids from the tissue matrix and improve the bioavailability of phenolic compounds [37]. Intake of AXOS containing cereal by subjects (ages over 50 years old) increased blood ferulic acid levels [31]. Ferulic acid inhibits LPS-induced inflammatory response through modulation of NF-κB in macrophage cells *in vitro* [38]. It cannot be ruled out that the presence of increasing ferulic acid by consumption of enzymatic processed wheat bran may contribute to the anti-inflammatory effects of enzymatically-treated wheat bran. Future study is required to determine whether the anti-inflammatory effect of wheat bran is mediated by its prebiotic action or ferulic acid.

## 5. Conclusions

In conclusion, the wheat bran preparation we used is a relatively crude form prepared using only extrusion and enzymatic treatment without complex processing, such as removal of starch and protein. Following this simple process, wheat bran enhanced macrophage activity and exhibited anti-inflammatory effects *in vivo*. Our results provide evidence for the usefulness of enzymatically-processed wheat bran as a functional food with immunomodulatory activity.

## Figures and Tables

**Table 1:** Dietary fiber and soluble arabinoxylan in different wheat bran preparations.

Group	Total Dietary Fiber (%)	Soluble Dietary Fiber (%)	Insoluble Dietary Fiber (%)	Water Soluble Arabinoxylan (%)
WB	43.75 ± 0.15 ^b^	4.62 ± 0.21 ^a^	39.44 ± 0.13 ^c^	0.32 ± 0.14 ^a^
XWB	46.49 ± 0.19 ^c^	4.98 ± 0.16 ^c^	38.65 ± 0.12 ^b^	0.71 ± 0.17 ^b^
EXWB	38.65 ± 0.11 ^a^	4.85 ± 0.14 ^b^	30.84 ± 0.15 ^a^	7.14 ± 40.18 ^c^

WB: wheat bran; XWB: extruded wheat bran; EXWB: wheat bran prepared by extrusion and enzymatic treatment. Values with different letters in one column are significantly different (*p* < 0.05). Data represent mean ± SD (*n* = 3).

**Table 2:** Carbohydrate composition in various wheat bran preparations.

Group	Arabinose (mg/g)	Galactose (mg/g)	Glucose (mg/g)	Xylose (mg/g)
WB	0.04 ± 0.002 ^a^	0.01 ± 0.002 ^a^	0.23 ± 0.001 ^a^	0.08 ± 0.001 ^a^
XWB	0.06 ± 0.004 ^b^	0.01 ± 0.001 ^a^	0.30 ± 0.004 ^b^	0.10 ± 0.004 ^b^
EXWB	0.09 ± 0.004 ^c^	0.02 ± 0.003 ^b^	0.34 ± 0.001 ^c^	0.16 ± 0.002 ^c^

WB: wheat bran; XWB: extruded wheat bran; EXWB: wheat bran prepared by extrusion and enzymatic treatment. Values with different letters in one column are significantly different (*p* < 0.05). Data represent mean ± SD (*n* = 3).
